# Medico-Chirurgical Transactions, Published by the Royal Medical and Chirurgical Society of London

**Published:** 1839-01

**Authors:** 


### Art. VII.

*Medico-Chirurgical Transactions, published by the Royal Medical and Chirurgical Society of London.* Vol. XXI.—*London,* 1838. 8vo. pp. 450.

Neither the nature of this Society, nor the character of the writings hitherto published by it, demands from us any notice in this place: both are well known to the profession at large; and, as the contents of the present volume are such as to claim from us considerable attention, we shall, without further preface, proceed to their consideration.

I. The first article, by Mr. George Gulliver, assistant-surgeon of the Guards, is devoted to the enquiry—*Whether, in cases of Necrosis, the dead Bone admits of Removal by Absorption?* Morbid specimens in various collections, and experiments performed by the author himself, are alluded to, as giving a negative reply to the above question; or as, at least, tending to show with how much difficulty dead bone becomes subject to absorption, and that absorption cannot be regarded as the means by which the sequestrum disappears in cases of necrosis. This, it is observed, is contrary to the opinions of the best authorities on the subject. Of the facts brought forward by Mr. Gulliver the following is an example:

"A patient had anchylosis of the metacarpal bone with the proximal phalanx of the great toe. The articular extremities became necrosed, and several small sequestra were discharged, the disease having continued twenty-four years. The metacarpal bone was divided near its base with the cutting pliers, and the diseased bones removed; and the base was subsequently excised, to secure the anterior tibial artery. A blackened sequestrum, about the size of a horse-bean, was found in a cavity in the situation of the articulation of the phalanx with the metacarpal bone, where it had probably been inclosed for a series of years." (p. 9.)

There are other facts of the same kind alluded to. In the experiments of Mr. Gulliver, instituted with a view to establish the non-absorption of dead bone, small pieces of bone were kept in contact with the surfaces of ulcers and with the sinuses of setons, in the human subject, for periods varying from three to ten weeks. Pieces of bone were likewise introduced into the soft parts of the leg of a dog, into the subcutaneous cellular tissue of the same animal, and were allowed to remain a considerable time; no loss of weight being experienced. Other pieces of bone were introduced into the medullary canals of the tibiæ of rabbits, and were likewise, after detention for a considerable time, taken away unchanged.

We do not see the importance of many of these experiments as bearing on the points in question. With regard to the human morbid specimens mentioned, it may be said that they are exceptions to the rule; just as we find pins imbedded in omentum, and bullets encysted in various parts of the body and preserved in collections; although we never could infer from these that the rule was, for such and other foreign substances not to escape from the body by ulcerative absorption: and we cannot insist too much on the necessity of the greatest possible caution in drawing inferences from any analogical experiments. Can it be maintained that there is so far identity between the surface of an ulcer, or the lining of a seton, and the texture by which it is said that the absorption of sequestra is effected; that it is fair to infer from the fact that, if a piece of bone is placed in contact with either the ulcer or the seton, without any sensible diminution being effected in its weight, that *therefore* portions of necrosed bone cannot be removed by the absorbents? The experiments in which bone was inserted into the medullary canals are certainly more applicable to the question: but it may be said that, as these were performed on rabbits, and as the case was not one of necrosis, their results are by no means such as to enable us to form any very satisfactory opinion on the subject.

II. Mr. COPLAND HUTCHINSON has added some further remarks to those already communicated to the Society, *on the comparative freedom of sailors from calculous diseases.* With regard to the practical inferences derived from his observations, he adds,

"I have hitherto scrupulously forborne to offer any opinion on the treatment of calculous diseases, as the result of my extended statistical enquiry into the subject; but it may not be out of place for me here to state, that pure air, a lax state of the bowels,—iodine used internally in proper doses, and externally over the region of the kidneys,—the use of swings, either in a garden or elsewhere,—active bodily exercises, —warm clothing, such as flannel dresses worn next the skin,—and a very sparing use of vegetables, seem to me to be the remedial and preventive measures indicated in such cases, from a careful review of the statements contained in this and my former papers. One or more, or all of these, according to circumstances, may be advantageous, where sea-voyaging and a sea-life are impracticable." (p. 23.)

III. Dr. Bostock has laid before the Society a plan for arranging the phenomena observed in examining urine; hoping that, if information on the subject is obtained from many sources, arranged according to a definite plan, it may lead to important practical results. He wishes to engage the cooperation of such members of the Society as may think favorably of his plan; and we shall be glad to promote so desirable an object. The following are the headings under which he wishes the particulars of each case to be arranged:

"1. Designation; time; quantity.
"2. External characters; clearness; colour; odour.
"3. Specific gravity.
"4. Degree of acidity.
"5. Amount of solid contents per cent.
"6. Proportion of solid contents soluble in alcohol.
"7. Effect of heat.
"8. Effect of corrosive sublimate.
"9. Amount of precipitate by ammonia.
"10. Amount of precipitate by oxal. ammon.
"11. Spontaneous changes."

IV. Mr. Beaumont has described a new instrument for closing fistulæ between the bladder, vagina, and rectum, and fissures of the soft palate. He has aided the account of his instrument by a plate, which it is necessary to examine to well understand the description. It wants the sanction of repeated trials.

V. The next paper consists of *Facts and Inferences relative to the Condition of the Vital Organs and Viscera in general, as to their Nutrition in certain Chronic Diseases*, by Dr. John Clendinning.

In collecting the facts contained in this paper, the author has the ulterior object in view of proving, in a subsequent communication, the great frequency of diseases of the heart; "a class of diseases which," he imagines, "is the main cause of the great bulk, if not of the whole, of the sufferings and mortality ascribed by authors to numerous chronic diseases,—viz. to asthma; to chronic catarrh; to dropsy, including anasarca, ascites, and hydrothorax; to emphysema pulmonum; to chronic hepatic diseases, and to various affections denominated phthisis catarrhalis, catarrhus suffocativus, dyspnœa chronica, tussis senilis, winter cough of mature years, miliary tuberculation of the lungs, &c.; also of no small part of the gravity and fatality of acute diseases of all the great viscera in adult subjects, but more especially of those of the lungs, as well as of typhus and other continued fevers." (p. 36.) The proofs of the foregoing statement are to follow; and we hope that, in endeavouring to maintain his position, Dr. Clendinning will give us evidence as satisfactory as any such statement must require. The object of the present paper is to ascertain the modifications impressed on the nutritive functions in the viscera in certain chronic diseases. We must limit our remarks to the inferences which Dr. Clendinning considers himself justified in drawing from the numerous facts collected. He remarks, with much justice, on the uncertainty attending either visual or manual estimate of the weight or size of the viscera, and he agrees with Dr. Carswell that the best method of measuring parts in pathological enquiries is by weighing them. The following are Dr. Clendinning's inferences from the facts which he has collected, although he does not insist on the number of his facts being sufficient to render his inferences absolute:—1. The healthy adult male heart averages, for all ages under sixty, nearly eight ounces and a half avoirdupois. The healthy female heart averages seven ounces and a half, or, more exactly, seven ounces and two fifths. This agrees with the opinion of M. Bizot, mentioned in our eleventh Number, that, in every series of ages, the female heart is smaller than that of the male. 3. In phthisical subjects, the heart, in a large proportion of cases, weighs considerably more than in health. We know not how to reconcile this with the statement by Bizot, in the paper already referred to, "that, both in males and females, the heart is smaller in all its proportions, in tubercular subjects than in others." As the latter tends to contradict, so do the investigations of Dr. Clendinning tend to support the hypothesis of Laennec, Bertin, and Bouillaud, as to the agency of phthisis in producing hypertrophous heart. Our author appears, by having examined the weight of the viscus, to have instituted a mode of examination more satisfactory than that of measurement by the eye or rule; and a different mode of investigation may explain the want of uniformity in the inferences of the several investigators. 4. His fourth inference is in accordance with that of M. Bizot, that the weight of the heart increases with years up to the end of life, contrary to the law of nutrition of the viscera in general. 5. Hypertrophy of the heart generally, or of the left ventricle alone, predisposes not only to visceral or general plethora and hypertrophy, but also to acute and chronic inflammations in general, and especially to bronchitis, pneumonia, and pleurisy. 6. The average weight of the brain of the healthy adult male under sixty years of age is about 45·85 ounces; that of the healthy adult female under sixty, about 41·25 ounces. 7. The weight, and consequently nutrition, of all the viscera exceed the normal standard in all cases of phthisis, in which the heart is increased in bulk or weight.

VI. *Remarks on Malignant Diseases of the Skin of the Face*, by Cæsar Hawkins, constitute the next paper: it is a valuable one, and we shall therefore condense it for our readers. By malignant diseases Mr. Hawkins means such as essentially possess a new structure, and are capable of exciting a poisonous influence in one or more of these several modes: 1, on the *neighbouring textures;* 2, upon the *absorbent system;* 3, upon the *whole constitution*. By this restriction of the term malignant are excluded, 1, the irritable ulcer described by Mr. Earle in the twelfth volume of the Society's Transactions; 2, the various forms of scrofulous disease which attack the nose, eyelids, and cheeks; 3, the several varieties of tubercular sebaceous disease; 4, hypertrophy of the skin of the nose. Malignant fungoid diseases, "in their *hematoid, medullary*, or *melanoid* varieties," seldom occur in the skin of the face until they have been formed in other parts: but the class of scirrhous or cancerous complaints are very peculiar when occurring in the skin of the face, and differ in many respects from what is usually called cancer. Cancer of the skin of the face is seen in three different forms.

1. *The common cancer of the face.* With this, as it shows itself in the lower lip, most surgeons are familiar: it commences generally by a little hard tubercle in and beneath the cutis, the ulcer which takes place in it being covered from time to time by a thin scab. A deep excavated ulcer succeeds, with characters which it is unnecessary to describe. When *common cancer* occurs in other parts of the face, it presents the same characters as when occurring in the lip. Complete removal of the whole of the diseased structure is well known to be the only remedial means which offers any hope of getting rid of the disease.

2. The second form Mr. Hawkins designates "*cancerous ulcer,*" or "phagedenic ulcer of the face of old persons." He believes its usual origin to be a flat, brownish tubercle, generally situated in the angle between the cheek and ala nasi, or in the inner canthus of the eye, which is frequently stationary for a long time before some accidental violence induces ulceration: this tubercle is softer, flatter, and darker than that of common cancer, as if it implicated the outer texture only of the cutis, including the coloured rete mucosum. The ulcer has a dark shining appearance, with slight elevation of its edges, which are jagged and irregular, and the skin around it is not thickened nor inflamed as in ordinary cancer; from the ulcer of which it is also distinguished by the trifling pain which accompanies it, by the absence of hemorrhage, sloughing, and fungus, and by its very slow progress; many years sometimes elapsing before very extensive ravages have been committed by it, during which time the ulcer sometimes remains nearly stationary for a time, or becomes covered by a thin skin, in which the vessels of the subjacent texture are visible; and in these intervals of rest the new structure at the edges diminishes in thickness. In a more advanced stage of the disease, when the ulcer has opened into the cheek or malar and maxillary bones, its difference from ordinary cancer is evinced in the most remarkable manner, by the little disturbance which it causes in the general health, and by the entire absence of contamination (as far as the author is aware) in the absorbent glands. For this disease Mr. Hawkins prefers the term *cancerous ulcer* to lupus or lupoid tubercle, as it is sometimes termed; as indicating the scirrhous nature of a new structure, possessing a malignant influence upon every texture in its neighbourhood, yet inferior to common cancer in the degree of malignancy, since it does not contaminate the absorbent glands. Mr. Hawkins considers that removal by the knife of either the tumour or the ulcer is the best treatment; but, in a broad flat ulcer, without any depth of new structure, he employs the chloride of zinc, which he has frequently used without any of the injurious effects of other caustics.

3. The third form may be called the *cancerous tumour*, or *fungous cancer of the face of old persons*, and Mr. Hawkins is not aware of its ever having been before described. Its early stage is a small round or oval tumour in the skin, generally in the cheek, or over the malar bone, or on the ala nasi. It is nearly of the natural colour of the skin for a long time, or is a little whiter, from the outer part of the cutis being thinned by the growth of the tumour, so as to allow the colour of its interior to shine through it. When cut, the tumour appears white, solid, but not very firm, with a well-defined margin, separate from the rest of the skin, and where it projects below the cutis it is covered by a kind of cyst. The tumour is more globular, soft, insulated, and distinct, more completely confined to the texture of the skin, more elevated and less liable to become puckered than ordinary cancer of the skin of the face, and less liable to have lancinating pain before the ulcerative stage has begun. It is more elevated and circular, of a whiter colour, more abrupt at its margin, and extends deeper into the substance of the cutis than the tubercle of the *cancerous ulcer*. It has fewer vessels ramifying on its surface, and has none of the livid colour previous to its ulceration, of fungus hæmatodes, nor of the darkness of structure of melanosis, and its texture is firmer and more organized than that of medullary tumours. It is distinguished, too, from all these by its being single, and by the length of time that it remains stationary. If it forms upon the nose, it is easily distinguished from the tumours of hypertrophy of the ala nasi by the absence of surrounding redness and thickening, by its defined cyst-like limits, and by its having none of the enlarged sebaceous follicles observed in that disease. The tumour grows thus smooth, globular, and nearly unattended with pain, to the size of a nut or of a walnut, before it excites apprehension in the patient's mind. At last it is pricked or irritated, or ulcerates spontaneously, and there arises a mass of healthy granulations from the surface, which spread out considerably beyond the tumour, over the surrounding skin, to the height of an inch or more, with a copious discharge of healthy pus, without fætor and without sloughing or bleeding, and not even now very painful. The tumour at the basis of these granulations increases in depth and in diameter, but is free for a long time from any attachment by altered cellular texture to the subjacent parts, so as still to allow of removal with every chance of success. The circular prominent fungus of this disease appears very different from the soft irregularly formed granulations and the excavated ulcer with everted margins of the *common cancer*, and from the flat, dark-coloured surface, destitute of granulations, of the *phagedenic ulcer;* neither has it any resemblance to the bleeding sloughing surface of *fungus hœmatodes*. The tumour grows to a considerable size before it alters its character, and before the general health suffers much. After a time ulceration extends more deeply into the tumour, and its projecting appearance is lost; the bones and deeper parts become rapidly changed into the new structure, which in some parts is gristly like scirrhus, but in others is softer and more pulpy, like some cases of medullary disease of the bones. The ulcer in this stage is also somewhat intermediate in character between these two diseases. In its third stage of advanced ulceration, the cancerous tumour becomes more like common cancer of the lips and face; but there is more tumefaction round and beneath the ulcer; the edges are less curled and hardened; the discharge is healthy purulent secretion, instead of offensive watery and sanious fluid of a peculiar odour; and there is much less disposition to bleeding and sloughing. In malignancy it is intermediate between the cancerous ulcer and the common cancer; admitting therefore of removal by the knife, if sufficient care be taken to excise the whole, with more chance of the cicatrix remaining sound than in ordinary cancer; in fact, with almost a certainty of success, where it has not attained a great magnitude.

VII. Mr. G. Malcolmson has communicated a paper *on a peculiar Symptom occurring in some Cases of enlarged Liver,* which he describes as "a loud sound (as heard through the stethoscope), between a crepitous rattle and a bleating, and accompanied by a vibration of the parietes of the thorax, communicated to the hand applied to the part." In another part of the paper this sound is spoken of as "partaking of the character of a bleating and of an ordinary respiratory murmur." In one case it is said that such a sound was caused by the thin edge of the lung being compressed against the costal pleura by an enlarged liver. The same symptom, it is observed, occurs from simple enlargement of the liver; and a knowledge of it may be of use, both in relieving the mind of the sufferer and in directing the practice in certain obscure cases. Mr. Malcolmson mentions a case of enlargement of the liver, where he was able, "by placing the patient in a sitting posture, to remove the symptom, and to let the lung descend a little further into the chest, and, by pressing the liver forcibly upwards, again to produce it."

We regret that we have not space for some valuable remarks on hepatic abscess, but must refer our readers to the volume. We are happy to find the author recording his opinion of the injurious effect of employing mercurial medicines when it is known that abscess in the liver has taken place.

VIII. Dr. John Wilson has described various cases of *Nervous Affections peculiar to young Women, causing contraction of the muscles of the extremities, accompanied by increase, diminution, or absence of sensation or motion.* "The cases have been taken from the case-book in the order in which they occurred, without any selection, except in omitting those cases of less severity which, in general, much sooner terminated favorably." They will not admit of abridgment with advantage. They belong to diseases very little understood; and to acquire an acquaintance with which is, from the moral as well as physical nature of the subjects of them, a very difficult task. Dr. Wilson, perhaps wisely, attempts to form no theory for their explanation; and his treatment, on the whole, appears to be guided by no principle but perseverance. "In the cases related the women were all young, (from eighteen to twenty-six years of age,) generally in good health and of strong constitutions; all of them were single, and many of them subject to violent hysteric fits. In the four most obstinate cases the functions of the uterus were regular; in others the bowels were confined, and in four of them obstinately." Any one familiar with the female wards of a large hospital will recognize the cases to which the following symptoms belong: Pains, of various kind and degree, in all parts of the body, fixed and shifting, attended sometimes with the greatest sensibility to the least touch; affections of sight; tinnitus; various degrees of affection of the muscular system, paralysis, contraction of the limbs, and stiffness of joints, with or without change of common sensation; symptoms more commonly regarded as hysteric, some or all of which symptoms have been treated by copious bleedings, leechings, and blisterings, without any or with but temporary relief. To these various symptoms, occurring in young females, Dr. Wilson has applied the following means. We give them "en masse," because, excepting where, as in the case of purgatives, the indication was obvious, it is not evident on what principle much of the treatment was employed: Acupuncture, moxas, blisters, sinapisms, euphorbia plasters, cupping, cold shower-baths, douches, purgatives, turpentine enemata, creosote, hydrocyanic acid, iron, forced extension of the contracted limbs, kicking, &c. &c. Dr. Wilson remarks on the frequency with which these nervous females mistake the causes of their symptoms; and it is unnecessary to repeat what he says of their various tempers during the progress of treatment. We recommend his paper to an attentive perusal; but these cases, of all others, are perhaps the least to be understood from books.

IX. The next paper is an unquestionable instance of *a secondary occurrence of Measles in a child*, æt. 22 months, three months after the first attack; the symptoms being in each case unequivocal. The case is reported by Dr. Joseph Moore.

X. Mr. Travers has lately removed the *Clavicle with a Tumour situated in it.* The case is of a boy, æt. 10, on the centre of whose collar-bone a firm but not painful swelling, the size of a hedge-nut, was discovered in the summer of 1836. It was supposed to have originated from a blow received about ten days previously. Leeches and cold lotions were used, but the tumour grew, and, when Mr. Travers first saw it, was oval-shaped, about as large as a pigeon's egg, firm but elastic, and painful only when compressed. It gave the idea of a false joint after a central fracture, or at least of a cyst enclosing the broken and ununited portions of the bone. The tumour slowly increased in spite of remedies. May, 1837, the base of the tumour, from its scapular extremity, occupied full three fourths of the bone; about two-thirds of its circumference was supra-clavicular, so that, in the erect position of the body, it was seen by a person standing behind the patient over the fall of the trapezius. The skin had a purple hue, from a congestion of the superficial purple veins, but there was no sign of pressure on the blood-vessels or nerves of the arm. About twelve months from the date of its commencement the disease was removed, and the operation is thus described:

"The little patient being recumbent, with his shoulders raised and head slightly averted, a crucial incision was made through the integument and platysma myoides, one limb of which was nearly in a line with the clavicle and the other at right angles; and the flaps and facial coverings successively dissected down to the external basis of the tumour. The pectoralis and deltoid muscles were then carefully detached from their clavicular origin, avoiding the cephalic vein, and the fibres of the trapezius and cleido-mastoid muscles divided on a director. One considerable vessel, in the situation of the transversalis humeri, required a prompt ligature. The circumference of the tumour was now well defined, though it was found to be firmly imbedded, and adherent on its posterior aspect. Disarticulation of the scapular extremity of the bone was next effected without difficulty, and the mobility thus communicated to the mass facilitated the completion of the operation. A director was now worked beneath the bone, as near to the sternal articulation as was practicable, and, with a pair of strong bone-nippers thus introduced, it was completely and clearly divided. The subclavius muscle and a part of the rhomboid ligament were now detached from the tumour, and the mass being well raised by an assistant, while the edges of the wound were kept wide apart by metallic retractors, the cervical prolongations of the tumour were separated from their remaining connexions by a few touches of the scalpel, without injury to the subclavian vessels. The operation occupied some time, but the boy displayed good courage as it proceeded: very few vessels were tied, and the loss of blood did not exceed twelve ounces." (p. 137.)

Recovery was very satisfactory. There is scarcely any perceptible falling forward of the shoulder, nor any restriction of the motions of the arm: he elevates it perpendicularly over his head, extends it horizontally, carries and rotates it behind the trunk, and performs the same extent and variety of circumduction, and with equal promptitude and power as the parallel movements of the other arm. The production of bone from the truncated sternal extremity of the clavicle extends at least two inches, and terminates beneath the centre of the cicatrix in a firm ligamentous band adherent to the skin.

The tumour presented anteriorly a regular curvilineal surface, posteriorly it was irregular. A dense fibrous expansion invested it on all sides, and, from the puncture of the principal cyst during the operation, a dark grumous fluid escaped. Its interior was composed of cells of nearly equal dimensions, filled with dark, solid coagula of blood; the scalpel grating, as it passed, upon particles of osseous matter. One larger cell was without a clot, having been filled with the dark fluid blood before mentioned. The investing membrane was condensed periosteum, the cells were irregularly expanded cancelli, and the calcareous particles were the debris of the bony plates and walls. The occurrence of the tumour Mr. Travers is disposed to ascribe to the blow.

There are four other cases of removal of the clavicle on record; one for caries, and three for osteosarcoma. All were attended with a successful result; one only having died of pleurisy, consequent on exposure too soon after the performance of the operation. We cannot forbear quoting the following interesting remarks, which arise out of the subject of injury to bone.

"There can be no doubt that injuries, more serious in their consequences than those of fracture and displacement, are often inflicted upon bone; as when adhesive (i.e. ossific) inflammation, the only healing process of which bone is capable, is prevented by the circumstances of the injury; or, on the other hand, is set up in preternatural situations or in morbid excess. Sometimes a bone is killed outright, sometimes disorganized only in part, by a blow or fall; and the particular circumstances which in such cases determine the separated periosteum, or the deranged cancellary membrane, to secrete bone or to secrete pus, to generate osteo-sarcoma or the medullary fungus, are not merely local but constitutional. The differences which induce in one case interstitial ulceration (caries), in another progressive ulceration or detachment from the living margin (exfoliation), in a third a process of renovation simultaneous with the disorganizing process, or a transfer of the nutrient action from one set of secreting vessels to another, at the expense of the original structure (necrosis): all these differences lie open for investigation under the general head of diseases of the bone from mechanical violence, apart from section, fracture, and displacement. They have hitherto escaped description, if not notice, under this head, being for the most part produced by causes apparently so inadequate and indirect as to evince a morbid state of the constitution in the individual. The following categorical summary I think my own experience will enable me to verify; the records of surgery will do so abundantly. 1. Periosteal inflammation and bony accretion or deposit upon the surface of the bone, with and without previous suppuration. 2. Inflammation terminating in abscess of the periosteum or cancelli. 3. Inflammation and ulcerative interstitial absorption of the bone (caries). 4. Rupture of the periosteal vessels and partial absorption of the bone, or rupture of the blood-vessels of the cancelli, and extravasation and absorption of the entire bone. 5. Osteo-aneurism, or aneurism of the capillaries within the bone. 6. Death and exfoliation of a portion of the bony shell. 7. Disorganization and separation by ulcerative progressive absorption of the entire shaft, after the deposit of a periosteal shell (necrosis). 8. Disorganization and death of a defined portion of the entire cylinder of the long and entire substance of the flat bones, and consequent external abscess. 9. Exostosis, periosteal or medullary, or osseous tumour of the long and flat bones. 10. Spina ventosa, or inflammatory and sanguineous deposits in the cells of the articular extremities of bones, irregularly expanding them, and ultimately reducing the walls of the bone to a network, crackling upon compression. 11. Osteo-sarcoma, or cartilago-osseous tumour of the periosteum and surrounding textures, secondarily implicating the bony and contiguous soft structures. 12. Malignant or medullary and hæmatoid fungus of the cancelli, and deposit of new septa or new exterior shell." (p. 145.)

XI. Mr. Ancell has described *a Case of universal Purulent Deposition into the Joints, with Separation of the Epiphyses; occurring as a sequel to Small-pox.* It is a well-marked case of a rare disease.

XII. The next communication is *a Report of Twenty Cases of Malignant Cholera, occurring in the Seamen's Hospital-ship, the Dreadnought, between the 8th and 28th of October*, 1837; by Dr. Budd and Mr. Busk. To give at all a useful analysis of this paper would occupy so much space that, however reluctantly, we must pass it over.

XIII. The next is *on Aneurisms of the Heart, with Cases,* by John Thurnam. This is a very valuable and elaborate essay on the subject, the author having derived his information from every accessible source which appeared to be deserving of credit. There appears to be no instance on record of the right ventricle being the seat of aneurism; a fact which has been variously explained. The less perfect closure of the auriculo-ventricular opening on the right than on the left side (a fact which has been long admitted) will serve, in part, to account for the immunity from aneurism possessed by the right ventricle: but, in addition to this anatomical or mechanical peculiarity, Mr. Thurnam considers that, from the right cavities of the heart being the centre of the venous system, and partaking of the characteristics of that system, they cannot be the seat of aneurism: and, in support of this view, the author mentions it as a remarkable fact, that there is not on record a single authentic case of lateral or sacculated aneurism of the pulmonary artery. A definition of aneurism, founded on the above views, is as follows: "*an abnormal dilatation of a portion of the vascular system of red blood, either dependent upon or necessarily connected with a morbid change in the tissues forming the walls of the dilated part.*" This definition excludes from the class of aneurisms all forms of dilatation of the right cavities of the heart and of the pulmonary artery, and all general dilatations of the left cavities. We agree in the inapplicability of the term aneurism to any general dilatation of any of the cavities of the heart, although we are not satisfied with the reasons why the right ventricle should not be the seat of aneurism. The less perfect closure of the tricuspid than of the mitral valve; the less force required by the right than the left ventricle in propelling its blood through the pulmonary circulation; and perhaps the opinion of M. Breschet, that the apex of the right ventricle is stronger, in proportion to its lateral walls, than that of the left, and consequently better able to resist distension;—one or more of these afford sufficient anatomical reason, apparently, for the immunity of the right ventricle from aneurism, without referring it to any hypothetical vital peculiarities.

The history of the disease is derived by Mr. Thurnam by the application of the numerical method to fifty-eight cases. It is met with under two principal forms, its size varying from that of a nut to the size of the heart itself; the opening from the ventricle into the aneurismal sac consisting of dense fibrous tissue, well defined. The sacs are variously formed; sometimes of muscular substance and pericardium, sometimes (though but rarely) of endocardium and pericardium alone, sometimes of all the structures composing the ventricle. These sacs are susceptible of various kinds of degeneration; such as the steatomatous, cartilaginous, calcareous or osseous; and they not unfrequently are adherent to the fibrous pericardium. The contents are commonly coagula of various forms, or the sacs are found to be empty. The thinnest parts of the ventricular walls,—that is to say, the apex and highest part of the base of the heart,—are those which become much more frequently than others the seats of aneurism. Cases occur in which several aneurisms coexist. The pathological changes most often found together with aneurism are such as are almost universally regarded as effects of inflammation. Hypertrophy and dilatation are likewise found with it. The disease occurs much more frequently in the male than in the female; and after adult age it does not appear to be confined to any particular period of life. There is very little which is at all satisfactorily known of the causes of the disease. In regard to the nature of the disease, Mr. Thurnam says,

"From an examination of the anatomical details, as well as of the apparent causes of the disease, I come to the conclusion that, in twenty-two cases out of the fifty-eight, the aneurism originated in a dilatation of all the structures entering into the composition of the walls of the heart; and in six in a solution of continuity of the lining membrane and inner stratum of muscular fibres, either as a consequence of ulceration, or, what is more probable, of rupture; whilst, in the remaining thirty cases, the disease was either too far advanced, or the data given are insufficient to enable us to form a satisfactory opinion on this question. I therefore conclude that this lesion, in by far the greater proportion of cases, is of the nature of *true aneurism;* or that it has its origin in a dilatation of a portion of the walls of the heart, which has become less able to resist the distending force of the blood during the ventricular systole, in consequence of organic changes in the tissues composing it. In a great majority of instances, these changes would appear to have been the result of a more or less active antecedent inflammation." (p. 228.)

A more or less decided enlargement of one of the natural interspaces between the smaller fleshy columns of the heart, is mentioned as the earliest stage of those morbid changes which terminate in the formation of true aneurism. In one case, the author "met with a small cavity in the centre of the interventricular septum, which was capable of containing a small horse-bean. This cavity was evidently an enlargement of one of the natural sulci, which have been alluded to; it was traversed by the lining membrane of the heart, which in this particular spot was white and opaque, and it was only separated from the cavity of the right ventricle by a very thin stratum of muscular fibres, of a whitish appearance, and dense fibrous texture." (p. 229.) This observation is interesting and important; and is applied by the author to explain the mode of occurrence of biloculate and multiloculate aneurisms. Although regarding by far the greater number of cases as being instances of true aneurism, Mr. Thurnam believes that the existence of false aneurism of the heart is indubitable, originating in partial rupture, and perhaps (although this is not supported by any case) in ulceration, and in the discharge of the contents of abscesses and cysts into the cavity of the ventricle. In reviewing the various forms of cardiac aneurism, Mr. Thurnam concludes, that, with the exception of the external mixed aneurism, for the non-occurrence of which there is an anatomical cause, we find in the heart, all the varieties of the disease, which are met with in the arteries themselves. It is sufficient to say, that the symptomatology and diagnosis of this affection are entirely unknown, and that, under any circumstances, nothing but a probable guess could be made of its existence.

"In twenty-four cases, the mode of death is stated. In twelve of these, in which it was very sudden, it arose, in three from syncope; in one from an unknown cause; and in eight from internal hemorrhage. In six of these eight cases, the hemorrhage was dependent upon a rupture of the aneurismal sac into the pericardium; in one, upon a rupture of the sac into the left pleura; and in another upon a rupture of the substance of the ventricle itself, in the immediate neighbourhood of the sac. In four cases, the patients appear to have died from an apoplectic or paralytic affection, and in one from epistaxis. In three cases, the mode of death was by asphyxia, and this was probably the case in six other instances. In six cases, as well as in the four apoplectic cases, death was evidently the result of complication with other diseases." (p. 237.)

Aneurism occurs in the left auricle, but much less frequently than in the corresponding ventricle. The valves of the heart are also sometimes the seat of dilatations, "which may properly enough be styled aneurismal." To these we cannot at present do more than make the above reference.

XIV. *History of a Female who has four Mammæ and Nipples*, by Dr. Lee. We noticed this case in a former Number, Vol. V. p. 585.

XV. Dr. John Wilson's paper, on *the Results of Poisoning by Sulphuric Acid* is short, and contains only two cases: they have not much novelty.

XVI. If subsequent experience should confirm Mr. Henry Hunt's observations *On the Use of Arsenic in some Affections of the Uterus*, he will have the merit of calling the attention of the profession to a valuable remedy. Mr. Hunt's attention was first attracted to the subject of the influence of arsenic on the uterus by the pain attending a case of cancer uteri having been greatly relieved, when the constitutional effects of arsenic were produced in the system, by the use of the liquor arsenicalis. The physiological action also of arsenic on the generative organs further called his attention to this remedy, and afforded him a means of explaining the action of the medicine in disease. Various forms of diseased function of the womb appear to have been much relieved by the continued use of arsenic. Several cases are related and are well worthy of perusal. The first of them are cases of irregular and profuse menstruation, without inflammation or organic disease of the uterus, the excessive flow of the uterine discharge appearing to be the consequence of exhaustion.

Immediate and progressive improvement followed the use of the arsenic; and that such improvement was fairly attributable to the medicine is inferrible from the fact, that in three cases, the disorder had previously resisted every remedy that had been employed: in another case, "the immoderate discharge was arrested whilst taking the arsenic the first time, but returned soon after it had been left off, and was again immediately and permanently checked by resuming it." We think the following case well worthy of attentive consideration; knowing the difficulty which frequently attends all attempts to treat successfully cases of such a character.

"Mrs. Burne, æt. thirty, married but never pregnant, was labouring under the following symptoms in June 1837. A constant pain with heat, varying in severity, in the left groin and under the pubes, and bearing down, which was much increased by walking, standing, or sitting upright, by a costive state of bowels, or by the action of purgative medicine. She was easier in bed or reclining on a sofa, her urine was sometimes high coloured, at other times pale, her pulse rather quickened. On examination, per vaginam, the uterus was found to be tender and rather tumid. She attributed her disorder to the menses having been suddenly checked by exposure to cold three years before. She had consulted many medical men, who had bled her generally and locally, and always with temporary relief; purgatives, opiates, the warm and cold baths, and numerous other remedies having been employed without any lasting benefit. Thinking this to be a case of chronic inflammation of the uterus, I confined the patient to her bed, and, for six weeks, kept her under the influence of a mild course of mercury, with nitre and colchicum. While she remained in bed she was easy, but the pain returned with equal severity immediately on her leaving it and taking exercise. I then sent her home, and directed a pill with one twentieth of a grain of arsenic to be taken three times a day, which she continued four months, at the end of which time, the pain, which had gradually decreased after she had taken the pills six weeks, entirely ceased. She now attends to the active duties of a bake-house, and only suffers pain about the time of her menstruation. This case may perhaps be considered similar to those described by Dr. Gooch, as the irritable uterus." (p. 283.)

The next case described is one of neuralgia of the face, occurring at the menstrual period, and apparently connected with irritation of the uterus. The neuralgia was relieved by the arsenic, as is frequently the case with neuralgia quite unconnected with uterine disturbance. The cases related by Mr. Hunt, appear so certainly to indicate that arsenic possesses a beneficial action in certain morbid conditions of the womb, that we have much pleasure in being the means of bringing it more under the notice of medical men, hoping that it may be found of more extensive applicability than to the cases mentioned by Mr. Hunt. The quantity of arsenic given at first by Mr. Hunt is one twentieth of a grain of arsenious acid, and this in the form of a pill, three times daily. He finds that the stomach has much greater tolerance for arsenic when administered as a pill than in the form of Fowler's solution, and "the most sensitive, by taking the pill immediately after meals, have been enabled to continue it as long as it has been necessary, while others can take two pills or one tenth of a grain, three times a day, for a considerable period, without any unpleasant effect."

XVII. Mr. Perry has communicated a remarkable and interesting case of *Excision of the entire Lower Jaw;* the operation being rendered necessary by necrosis following an unusual course.

XVIII. In a paper on *Concentric Hypertrophy* of the heart, Dr. Budd has taken great pains to ascertain whether the condition to which the term is applied is an actual disease, or whether the opinion entertained by M. Cruveilhier, of its depending on the mode of death, is the correct one. We have only space for the conclusions to which his examinations of several cases have led him. I. In the recorded cases of concentric hypertrophy of one of the ventricles unconnected with valvular disease, there was no permanent diminution of the cavity during life; because, 1, similar appearances have been observed in the hearts of persons who died by the guillotine, and in subjects who died by cholera; 2, in these cases, the symptoms of cardiac disease were slight, not such as must have been caused by a ventricle so much contracted as it appeared to be after death; 3, in two cases, the cavity was restored by mechanical means to its normal size, and in none was there any obstacle behind it, by which its permanent diminution could be explained. II. In six cases, complicated by extensive valvular disease, the diminution of the cavity cannot be explained by an obstacle behind it; and in some of these cases, the existence of an obstacle before it renders it highly probable that this diminution was merely a passing condition of the ventricle; and as the appearances of concentric hypertrophy were not more marked in these cases than in those of the former class, and as the symptoms of obstacle to the circulation, observed in these cases, were such as would result from the diseased valves alone, we cannot admit the existence of concentric hypertrophy in this class. III. Concentric hypertrophy of a ventricle (mostly the right) in a high degree, with obstruction at its discharging orifice and an extraordinary passage for blood, occasionally exists as a congenital malformation. IV. Hypertrophy of the heart, to whatever extent it exists, when it is exempt from dilatation of its cavities, and from disease of the valves, does not produce any of the symptoms of an obstacle to the circulation through the heart.

XIX. Mr. Samuel Hadwen has related a *Case of Popliteal Aneurism*, in which hemorrhage succeeded to ligature of the superficial femoral artery, for which the femoral artery was tied immediately below Poupart's ligament. The hemorrhage from the first wound recurred, and as the ends of the bleeding vessel could not be discovered, the thigh was amputated. For nineteen days the case proceeded favorably, at the end of which time, the wound in the groin began to bleed, the blood being arterial and flowing in increasing quantity. The following day, as very profuse bleeding had greatly endangered life, a ligature was passed round the external iliac artery, in the manner recommended by Sir A. Cooper. On the twenty-ninth day after the operation, the ligature came away from the external iliac, and the convalescence of the patient was steady and permanent.

Experience confirms, (it is observed by Mr. Hadwen,) what the anatomical peculiarities of the femoral artery in the upper part of its course would indicate as highly probable, the danger attending ligature of the vessel in that situation; a danger, it appears, very far greater than that of passing a ligature around the external iliac artery. For "of eight cases in which a ligature was applied to this (the superficial femoral) artery, six were attended with consecutive hemorrhage, two with death, and two with a favorable separation of the ligature; giving to this operation a highly dangerous character." But Mr. Hadwen "cannot find a single case recorded of bleeding attending the separation of a ligature placed upon this (the external iliac) artery; so that it may be said, not merely as Mr. Hodgson observes, that the external iliac may be tied with as much safety as any artery to which a ligature has been applied, but that, of all the large vessels of the human body, it is the one that may be tied with the greatest security, as far as the effects of the operation are concerned, and with the best effect upon the diseases to which it is applicable." Mr. Hadwen's conclusion, and it appears to be quite justified by his fact, is, that except in a wound of the artery at the groin, ligature of the common femoral artery should never be performed.

XX. This is a *Case of Hydatid of the Liver successfully tapped*, by Dr. Cox, of Yarmouth.

XXI. We have in a previous Number given a notice of Dr. W. Thomson's first paper, *On Black Expectoration and the Deposition of Black Matter in the Lungs*. In the present volume is a second paper on the subject, which is devoted to the opinions entertained by medical authors on the disease in question. It is a very careful and elaborate compilation, well worthy of perusal, but not such as, with the limited space which we could afford to it, would supply matter of much interest to our readers.

XXII. Sir David Dickson next relates the history of *a Case of Enormous Ventral Aneurism*, which is only remarkable for its vast size. It originated in the descending aorta, and "was so immense that, with the exception of the cæcal region, it might be said to occupy the epigastric, both hypochondriac, the umbilical and left iliac regions, and the pelvis."

XXIII. Dr. G. Rees, in a paper, *on the Proportions of Animal and Earthy Matter in the different Bones of the Human Body*, has been led to the following conclusions:

"1. The long bones of the extremities contain more earthy matter than those of the trunk. 2. The bones of the upper extremity contain somewhat more earthy matter than the corresponding bones of the lower extremity, the difference being about one half per cent. 3. The humerus contains more earthy matter than the radius and ulna; and the femur more than the tibia and fibula. 4. The tibia and fibula contain, as nearly as possible, the same proportions of animal and earthy matter, and the radius and ulna may also be considered alike in constitution. 5. The vertebra, rib, and clavicle are nearly identical as regards the proportion of earthy matter, the ilium containing somewhat more of earths, the scapula and sternum somewhat less; the sternum containing more earthy matter than the scapula. 6. The bones of the head contain considerably more earthy matter than the bones of the trunk, but the humerus and other long bones are very nearly as rich in earths. 7. The metatarsal bones may probably be ranked with those of the trunk in proportional constitution." (p. 409.)

The above conclusions which are applicable to bones of the adult; likewise hold good in some respects with fœtal bones, for "the bones of the upper extremity (of the fœtus) contain somewhat more earthy matter than the corresponding bones of the lower extremity: the humerus contains more earthy matter than the radius or ulna, and the femur more than the tibia or fibula: the ilium contains somewhat more, and the scapula somewhat less earthy matter than the clavicle or rib." (p. 412.)

XXIV. The concluding paper by Mr. Tyrrell, is *on a successful Plan of arresting the Destruction of the Transparent Cornea from Acute Purulent Inflammation of the Conjunctiva;* a plan which its author characterizes "as one of extreme value in the treatment of this hitherto most destructive disease." It is a beautiful instance of the application of anatomical knowledge and accurate pathological principles to the treatment of disease; and we are induced by it to express a wish, which we have long entertained, that Mr. Tyrrell would benefit the medical profession by publishing his own rich and cultivated experience in ophthalmic disease generally. Amidst the mass of remedies which fall under our notice, it is no little relief to meet with one, the operation of which is so clearly and satisfactorily explained, and the effects of which are so decisive and beneficial. The plan followed by Mr. Tyrrell in this paper is, first, to "describe the mode of organization of the cornea; secondly, to explain how it is so rapidly destroyed in acute purulent ophthalmia; thirdly, to detail the mode of applying the remedy which has been found so successful, and lastly, to give some cases illustrative of its effects." With regard to the anatomy of the cornea, it is necessary to remember its inseparable union with the sclerotica; the closely adherent conjunctival covering, the existence of which as a distinct prolongation of the conjunctiva is chiefly seen in disease, when also it is seen that the chief vascular supply of the cornea is from the vessels of the conjunctiva, and not from the sclerotica.

The acute purulent inflammation of the conjunctiva, whether of idiopathic or specific origin, presents similar characters, runs through the same stages and leads to the same consequences, if unchecked. Among its well-known symptoms, we here allude to the great vascularity, to the chemosis either "complete or incomplete, as surrounding the margin of the cornea partially or entirely. When it is complete, (says Mr. T.) the cornea is in momentary danger by destruction of its vitality, which takes place in the following manner: the elevation of the sclerotic part of the ocular conjunctiva by subjacent deposit, renders it tense, and creates so much stress and tension on that part which is firmly bound down over the junction of the cornea and sclerotica, that the circulation through its vessels becomes impeded and ultimately arrested, so that the principal vascular supply of the cornea is cut off, and it dies or mortifies in part or in toto. The cornea first assumes a nebulous appearance, but its brilliancy remains; this, I believe to result from the deficiency of the interlaminar fluid, in consequence of impeded circulation; it resembles closely the appearance which may be produced by pressing the cornea firmly with a narrow body, so as to press the lamina together to exclude the interlaminar fluid. This nebulous state is usually general, and of short duration, and is succeeded by a dull and dense opacity of a part or of the whole of the cornea, which, at the same time, loses its brilliancy. There is not at any time evidence of any inflammatory action either in the cornea or in its conjunctional covering, and the destruction of these parts is usually too rapid to be the result of such morbid action in the textures themselves. The cornea then mortifies from a strangulation of its blood-vessels, and this strangulation is produced by the chemosis or elevation and tension of the conjunctiva which covers the sclerotic." (p. 420.)

Mr. Tyrrell's object in treating the disease was most effectually to relieve the tension of the chemosed conjunctiva, freely dividing it, without implicating its principal vessels; and he determined on the following mode of operation. He raised and secured the upper eyelid as far as possible, as in the operation for extraction, and then made free incisions into the sclerotic portion of the ocular conjunctiva, without injury to any other texture of the globe. He "considered it essential that the incisions should extend close to the margin of the cornea where the tension and pressure would be greatest, and that the direction of the wounds should correspond to the intervals between the insertions of the recti muscles, so that the principal vessels of the conjunctiva of the globe should not be injured."

The novelty in this communication is the mode of dividing the chemosed conjunctiva, and the anatomical reason for so doing. Mr. Tyrrell speaks of the disappointment and failure which attend on the ordinary method of dividing the chemosed conjunctiva, and if the principles here laid down are correct, the explanation of such failure is sufficiently easy. Several cases follow which should be attentively studied. They are the best evidence of the value of a mode of treatment which, it can only be lamented, has not been earlier known. From them we select the following, the first in which Mr. Tyrrell divided the chemosis, as above recommended. The case is that of a robust and strong young man with acute gonorrhœal ophthalmia in one eye. The palpebra were very much swollen, tense, and shining; the morbid secretion thick, copious, and of a deep yellow colour mixed with green; the chemosis was complete, and the cornea generally hazy or nebulous; but its surface was brilliant except at one point, where mortification had begun. The disease had not existed twenty-four hours. The chemosis was freely divided from the margin of the cornea towards the orbit once or twice in each space between the attachments of the recti muscles, the point of the knife being inserted just over the junction of the cornea and sclerotic, and passed outwards, the back of the knife being kept close to the sclerotic, as its acute edge divided the affected membrane: each incision had a direction radiating from the centre of the cornea. The chemosis was firm. Hot water was applied directly after the incisions, to encourage the bleeding. Soon after, the patient was bled from the arm, to the extent of about fourteen ounces, sufficient to relieve the fulness and firmness of his pulse: he took fifteen grains of calomel and colocynth, and was further directed to take calomel gr. ij., opii gr. ss., every six hours; to apply leeches freely to the palpebræ if pain recurred, and to bathe the eye frequently with a warm decoction of poppy. Diet of gruel, tea, toast and water, or soda water. On the next morning, the disease was found to be checked, the largest portion of the cornea had recovered its transparency, but an oval spot equal to about one third of the whole was dead: the chemosis and tumefaction of the palpebræ were much reduced; the conjunctiva was but lightly coloured, the morbid secretion was thinner and less copious, and he was free from pain; his medicine had acted freely, and he had applied a dozen of leeches to the eyelids on the preceding evening. In fortyeight hours after the division of the chemosed membrane, the acute stage was annihilated and he left off the calomel and opium. Recovery was rapid and perfect; there remained only a small, dense, opaque cicatrix in the cornea, which did not interfere with vision. Speaking of this case, Mr. Tyrrell says that, "I had not previously seen a single case in which the eye had been saved when the disease had made the same extent of progress." We wish that we had space for other of the cases, some of which show the admirable effects of the above treatment under circumstances far more unpromising than those of the above case. Other advantages attending Mr. Tyrrell's method are thus mentioned, "It renders unnecessary active depletion, or the adoption of any severe general or local measures likely to injure the general health, or to produce severe suffering. It cannot increase the risk of a case. It has been tried sufficiently to warrant my stating that it will be rarely found to fail; and I offer it with confidence to those, who, I feel satisfied, will give it a fair trial, and decide candidly and honestly on its merits." This paper is most creditable to Mr. Tyrrell, and cannot fail to add to his high reputation.

The notice which we have thus terminated of the last volume of the Medico-Chirurgical Society's Transactions, is almost purely analytical: by giving it this character, we have been enabled to present to our readers, the chief valuable practical matter which the work contains.

---

